# Research on health transition in Africa: time for action

**DOI:** 10.1186/1478-4505-9-5

**Published:** 2011-01-28

**Authors:** Dermot Maher, James Sekajugo

**Affiliations:** 1 Medical Research Council (MRC)/Uganda Virus Research Institute (UVRI) Uganda Research Unit on AIDS, PO Box 49 Entebbe, Uganda; 2 London School of Hygiene and Tropical Medicine, Keppel Street, London, UK; 3 Ministry of Health, PO Box 7272, Kampala, Uganda

## Abstract

With rapidly increasing globalization, trends towards unhealthy diets, obesity, sedentary lifestyles and unhealthy habits are resulting in an increased worldwide burden of chronic non-communicable diseases (NCDs). In Africa this means that health systems face the challenge of an increasing burden of NCDs and of continuing high morbidity and mortality from communicable diseases. This health transition represents an enormous challenge to Africa as the region with the least resources for an effective response. Whereas previous epidemics, including HIV, have caught Africa unprepared, the opportunity now arises to take the advancing wave of health transition in Africa seriously. Health research has a key role to play in meeting health and development goals, and must be responsive to changing disease patterns, such as health transition. There is an urgent need for research on health transition in Africa to enable countries to respond effectively to rapidly changing health needs.

Key areas of research include the following: epidemiological research so that a good understanding of the distribution in Africa of communicable and non-communicable diseases can inform health planning; research on the interactions between communicable and non-communicable diseases; health system research with a particular focus on new approaches to improve the primary care response to health transition; and policy research to evaluate the more upstream measures addressing the population-level determinants of NCDs. It is time to capitalise on the global policy environment, which is becoming more favourable to action on health transition in Africa, and implement a research agenda for health transition. Alliances have a key role to play in Africa as well as in other regions in implementing the research agenda on health transition by building research capacity and mobilizing the necessary investments.

## Introduction

These are exciting times for health research in developing countries, with increasing consensus on the role of research in meeting health and development goals [1,2]. The health research agenda needs to be responsive to changing disease patterns. Rapidly increasing globalization is accompanied by an increased worldwide burden of chronic non-communicable diseases (NCDs), including in developing countries. Sub-Saharan Africa faces particular challenges in responding to health transition, i.e. a double burden of communicable and non-communicable diseases. Health transition represents an enormous challenge to Africa as the region with the least resources for an effective response. Whereas previous epidemics, including HIV, have caught Africa unprepared, the opportunity now arises to take the advancing wave of health transition in Africa seriously. In this paper we highlight the urgent need for research to enable countries in Africa to respond effectively to rapidly changing health needs. Key research areas include the following: research on the interactions between communicable and non-communicable diseases; epidemiological research so that a good understanding of the distribution in Africa of communicable and non-communicable diseases can inform health planning; health delivery research with a particular focus on new approaches to improve the primary care response to health transition; and policy research to evaluate the more upstream measures addressing the population-level determinants of NCDs.

## Discussion

With rapidly increasing globalization, trends towards unhealthy diets, sedentary lifestyles and unhealthy habits are resulting in an increased worldwide burden of chronic NCDs (e.g. diabetes, cardiovascular and lung diseases, and cancer) and their associated risk factors (e.g. smoking, alcohol, hypertension, and obesity). While all low- and middle-income regions face the challenge of NCDs as increasingly important health problems, the African region faces the particular problem of an increasing burden of NCDs [3,4] and of continuing high morbidity and mortality from communicable diseases. Uganda provides an illustrative example of a resource-poor country in Africa in epidemiological transition. There is a continuing high burden of communicable diseases, with for example national estimates for 2007 of 5.4% HIV prevalence [5] and of tuberculosis incidence of 330/100,000 per year [6]. The importance of NCDs is increasingly recognized. The estimated national prevalence of diabetes was 98,000 in 2000 and is expected to rise to 328,000 by 2030 [7]. The prevalence of diabetes is particularly high in urban areas, with estimates suggesting that as many as 8% of the residents of the capital, Kampala, might have type 2 diabetes [8]. The estimated number of deaths in 2004 due to cardiovascular disease in Uganda was around 34,000 of which about 10,000 were attributable to ischaemic heart disease and around 12,000 to cerebrovascular disease [9].

Research on health transition is important because it imposes considerable costs on individuals, families and communities. Communicable diseases impose heavy human costs in terms of suffering and death, and also heavy financial costs on poor households. As NCDs become increasingly important causes of morbidity and mortality in developing countries, especially in Africa, they also incur high costs both to the individual and national economies [10]. Chronic NCDs have a huge negative economic impact and represent a significant impediment to human development in low- and middle-income countries [11]. In addressing inequalities in health in Africa, the agenda for research on health transition needs not only to maintain a focus on HIV and other communicable diseases but also to expand to include research on NCDs and on the interactions between communicable diseases and NCDs.

### Research on the interactions between communicable and non-communicable diseases

An increasing burden of disease in Africa arises not only from communicable diseases and NCDs but also from the interactions between them, e.g. between tuberculosis and malnutrition, human papilloma virus and cervical cancer, and hepatitis B virus and hepatoma. The burden of chronic NCDs (including for example diabetes) is likely to be further uncovered as scaled-up programmes of antiretroviral treatment (ART) of HIV-infected people lead to reduced HIV/AIDS mortality but increased morbidity related to chronic HIV infection and ART [12]. Increasing numbers of people in Africa are therefore at risk of possible metabolic side-effects resulting from life-long ART, but there is little knowledge of their epidemiology [13,14] or of how health systems can most effectively address them. Research on health transition in Africa needs to include research on the interactions between communicable diseases and NCDs: the biology of their interaction, their clinical course and distribution, and the delivery of interventions aimed at their prevention and treatment.

### Epidemiological research

Planning an improved health system response to health transition, with a priority for primary health care [15], requires assessment of the population burden not only of communicable diseases but also of NCDs and associated risk factors [16]. For example, the WHO STEPwise approach to surveillance of NCD risk factors uses a standard survey instrument and a methodology that can be adapted in different settings [17]. A lack of high quality health information is one of the major obstacles in developing appropriate preventative strategies for NCDs at the national level [18]. The need for good quality comparable data on disease burden and risk, to aid planning and implementation of prevention and control strategies for NCDs, is greatest among low- and middle-income countries [19]. There is an enormous gap between the sparse population-based data so far available on chronic NCDs and the comprehensive data needed for a good understanding of their distribution in Africa. Baseline data are needed, for example, on the prevalence of diabetes, hypertension and obesity as well as related cardiovascular disease risk factors in both rural and urban settings.

Prospective cohort studies are important in epidemiological research on health transition. They have made enormous contributions, for example, to our understanding of the dynamics and impact of the HIV epidemic [20]. In populations in developed countries, prospective cohort studies have proved crucial in understanding the aetiology, course and outcome of NCDs in have informed the design of prevention programmes, and are a valued long-term investment in public health. In Africa prospective cohort studies on NCDs and their risk factors can contribute to a better understanding of NCD aetiology, course and outcome [21]. In addition to the use of cohort studies to investigate a range of NCDs and their risk factors over the course of an individual's lifetime, long-term follow-up would also enable investigation of inter-generational health effects, e.g. the relationships between maternal health and nutrition status, intrauterine growth and susceptibility to cardiovascular and metabolic diseases. Although NCDs cause a large and growing disease burden in sub-Saharan Africa, there are very few prospective cohort studies on NCDs in developing countries in the region: the Birth to Twenty Cohort in Johannesburg-Soweto (South Africa), the Heart of Soweto Study on cardiovascular disease, and the Women's Health Study in Accra (Ghana) [22]. In meeting the urgent need for longitudinal cohort studies on NCDs in Africa, well established population-based study sites are ideal, since they offer cost savings [21]. The population-based cohort study in rural Masaka District in Uganda is a good example of how a population-based study site, in this case initially established for HIV epidemiology research, can be successfully used as a platform for research on health transition by expanded the research scope to include chronic NCD epidemiology [22].

### Health delivery research

Although research in Africa has made a substantial contribution to progress in improving the delivery of effective interventions against communicable diseases such as HIV, tuberculosis and malaria, research on NCDs has lagged behind that on communicable diseases. An effective response to health transition requires research on delivering effective interventions for NCDs as well as for communicable diseases. Cost-effective interventions for chronic NCDs are available [23] and successful primary care approaches to NCD care in developing countries have been developed (e.g. a model in rural South Africa) [24]. As the health service entry point for the vast majority of people, primary care is the key element in the health system response to health transition, playing a pivotal role in the delivery of prevention and care interventions for communicable diseases and NCDs [15]. In place of the current approach to primary care of people with chronic NCDs, which is often unstructured and inadequate, a programmatic public health framework for NCDs has been proposed [25] which builds on the experience of standardized diagnostic and management protocols and information management systems for HIV and tuberculosis interventions [26]. The response to health transition may be improved through the development of primary care services that cut across conventional categories of communicable diseases and NCDs. Health system research is needed on the effectiveness of these proposals for an improved response to health transition and on scaling-up effective approaches in large-scale delivery programmes [15].

### Policy research

Research on health intervention delivery programmes complements the more upstream, policy-oriented research priorities identified through a "grand challenge" exercise [27]. Evaluation of the relevance and effectiveness of these global policy research priorities is necessary in different settings, including in Africa as part of the overall research agenda for health transition.

## Conclusions

While global attention on health transition has largely been on India and China [28], we should take the advancing wave of health transition in Africa seriously. It is time to capitalise on the global policy environment becoming more favourable to action on health transition in Africa [29,30] and implement a research agenda on health transition which builds on the well established research agenda for communicable diseases such as HIV [31] and malaria [32] and on recent contributions to the agenda for NCDs [16]. Alliances such as the Global Alliance for Chronic Diseases [28] have a key role to play in Africa as well as in other regions by building research capacity and mobilizing the necessary investments [33].

N.B. The views of DM are not necessarily those of the Medical Research Council (UK).

## Competing interests

The authors declare that they have no competing interests.

## Authors' contributions

DM had the idea for the article which he developed in discussion with JS. DM took the lead in drafting the article and both authors contributed to the development of successive iterations. Both authors read and approved the final manuscript. DM is guarantor for the article.

## Funding

DM is the head of a research programme in Uganda funded by the Medical Research Council (UK).
